# The microbiological profile of nosocomial infections in an intensive care unit

**DOI:** 10.1186/1471-2334-14-S3-P73

**Published:** 2014-05-27

**Authors:** P Madhurima, G Sateesh, Y Saritha, M Padma

**Affiliations:** 1 Sentini Hospitals, Vijayawada, India

## Background

Nosocomial infections are responsible for morbidity and mortality in hospitalized patients. They also increase cost of treatment and prolong hospitalization. The aim was to study the nosocomial infections in intensive care unit (ICU) at tertiary care hospital.

## Methods

This study was done at a tertiary care hospital from June2012 to June 2013. Patients who developed infection after 48hours of stay in ICU were included in the study. Clinical samples were processed for bacteriological culture and susceptibility was tested using Kirby bauer disc diffusion method.

## Results

Of 2450 patients admitted to ICU, 237 (9.6%) suspected nosocomial infections were studied prospectively and total number of 302 samples were collected from all clinically suspected cases of nosocomial infections.

Samples included urine 91, blood 70, IV catheters 41, ET aspirations 61, ET tips 24, sputum 10 and Foley catheter tips 5. Out of these UTI, 96(31.71%) was the most common nosocomial infection followed by VAP84 (27.1%), BSI 55(23.1%) and catheter related infections 29(13.5%). Most common organism isolated was *K.pneumoniae* 78(32.8%), *E.coli* 53(19.6%), *A.baumanni* 44(18.5%), *S.auerus* 27 (11.3%), *P.aeuroginosa* 23(9.7%), *S. maltophilia* 12(5.06%). 41% of *S. auerus* were MRSA and all were sensitive to vancomycin.

## Conclusion

Strict infection control measures and antibiotic policy could reduce incidence of nosocomial infections.

